# Genomic association with pathogen carriage in bighorn sheep (*Ovis canadensis*)

**DOI:** 10.1002/ece3.7159

**Published:** 2021-03-02

**Authors:** Alynn M. Martin, E. Frances Cassirer, Lisette P. Waits, Raina K. Plowright, Paul C. Cross, Kimberly R. Andrews

**Affiliations:** ^1^ United States Geological Survey Northern Rocky Mountain Science Center Bozeman MT USA; ^2^ Idaho Department of Fish and Game Lewiston ID USA; ^3^ Fish and Wildlife Sciences University of Idaho Moscow ID USA; ^4^ Department of Microbiology and Immunology Montana State University Bozeman MT USA; ^5^ Institute for Bioinformatics and Evolutionary Studies (IBEST) University of Idaho Moscow ID USA

**Keywords:** Bighorn sheep, chronic carrier, family‐based genome‐wide association, genetic diversity, pneumonia, wildlife disease

## Abstract

Genetic composition can influence host susceptibility to, and transmission of, pathogens, with potential population‐level consequences. In bighorn sheep (*Ovis canadensis*), pneumonia epidemics caused by *Mycoplasma ovipneumoniae* have been associated with severe population declines and limited recovery across North America. Adult survivors either clear the infection or act as carriers that continually shed *M. ovipneumoniae* and expose their susceptible offspring, resulting in high rates of lamb mortality for years following the outbreak event. Here, we investigated the influence of genomic composition on persistent carriage of *M. ovipneumoniae* in a well‐studied bighorn sheep herd in the Wallowa Mountains of Oregon, USA. Using 10,605 SNPs generated using RADseq technology for 25 female bighorn sheep, we assessed genomic diversity metrics and employed family‐based genome‐wide association methodologies to understand variant association and genetic architecture underlying chronic carriage. We observed no differences among genome‐wide diversity metrics (heterozygosity and allelic richness) between groups. However, we identified two variant loci of interest and seven associated candidate genes, which may influence carriage status. Further, we found that the SNP panel explained ~55% of the phenotypic variance (SNP‐based heritability) for *M. ovipneumoniae* carriage, though there was considerable uncertainty in these estimates. While small sample sizes limit conclusions drawn here, our study represents one of the first to assess the genomic factors influencing chronic carriage of a pathogen in a wild population and lays a foundation for understanding genomic influence on pathogen persistence in bighorn sheep and other wildlife populations. Future research should incorporate additional individuals as well as distinct herds to further explore the genomic basis of chronic carriage.

## Graphical abstract

Bighorn sheep populations impacted by infectious pneumonia may experience long‐term recovery complications due to chronically infected individuals shedding pathogen and suppressing lamb recruitment in years following outbreak events. We investigated genetic associations with the chronic carrier phenotype in bighorn sheep to better understand underlying dynamics influencing pathogen persistence in wild sheep populations.

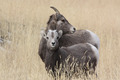

## 1 INTRODUCTION

Host susceptibility (vulnerability to infection), tolerance (ability to reduce the cost of infection), infectivity (ability to transmit infection), and resistance (ability to clear infection) influence transmission and persistence of pathogens in populations. Heterogeneity in these characteristics can produce a subset of individuals that contribute disproportionately to disease dynamics. For example, highly infectious individuals may act as superspreaders, resulting in disproportionately more new infections relative to other less infectious individuals (Lloyd‐Smith et al., 2005; Paull et al., 2012). Individuals with low resistance and high tolerance, or those with incomplete resistance, may act as chronic carriers (individuals that host and transmit pathogens for long durations) allowing infection to persist in populations. There is a growing body of literature assessing pathogen infection and host genomics in wildlife (e.g., DeCandia et al., 2020; Donaldson et al., 2017), and the influence of host genetics on host susceptibility, resistance, and tolerance has been widely documented (recent examples include Batley et al., 2019; Bishop & Woolliams, 2014; Oleński et al., 2019); however, few studies have investigated host‐genomic influence on chronic carriage.

Understanding the role of host genomics on pathogen carriage risk can provide insights for management of disease in humans, domestic animals, and wildlife. For example, in animals, genomic data can inform selective breeding for resistance to pathogens, which has been broadly discussed in agriculture and aquaculture (Bishop & Woolliams, 2014; Houston et al., 2020). These same approaches may be applied to selective breeding for individuals with low risk of becoming a chronic carrier. In free‐living populations, identifying individuals genetically predisposed to maintaining infection could inform future research as well as management actions such as culling, captive breeding, and translocations.

One approach to understanding the impact of host genomics on carrier status is through genome‐wide association (GWA) analyses. Genome‐wide association methods have been used to better understand the underlying architecture of disease (i.e., are few or many loci responsible for disease‐related phenotypes?) and to identify single nucleotide polymorphisms (SNPs) that may be associated with disease phenotypes (Bush & Moore, 2012). These methods have been successfully utilized to understand immunity and resistance in wildlife disease systems (Elbers et al., 2018; Kosch et al., 2019; Margres et al., 2018). While GWA methods are typically restrained to large sample sizes, they can be adapted for individuals with shared ancestry, including parent–offspring pairs and other relatives, through family‐based association designs (Ott et al., 2011), which can be useful when sample sizes are limited. Additional metrics assessing genome diversity, such as heterozygosity and allelic richness, can complement association analyses, identifying broader trends in diversity that may play a role in disease phenotypes.

Introduced disease has played a major role in reducing abundance and inhibiting recovery in bighorn sheep (*Ovis canadensis*) populations across western North America. Specifically, pneumonia epizootics (primary etiological agent *Mycoplasma ovipneumoniae*) have caused all‐age die‐off events of varying severities (48% mortality on average), with the most extreme resulting in 100% mortality and localized extirpation (Cassirer et al., 2018). Following outbreak events, *M. ovipneumoniae* may persist within a population via persistently infected and shedding individuals (often, asymptomatic; Plowright et al., 2017). Continued shedding by these chronic carriers often results in seasonal lamb exposure and mortality (20%–100%), suppressing recruitment and recovery in years following epizootics (Cassirer et al., 2018; Cassirer & Sinclair, 2007). These dynamics can result in ongoing population declines and stagnation with occasional herd extinctions (Manlove et al., 2016). It is recognized that *M. ovipneumoniae* carriers have a significant impact on herd recovery (Garwood et al., 2020); however, factors that influence an individual's ability to clear infection or transition to chronic carrier status are not well understood. Recent research suggests that paranasal sinus tumor presence in Rocky Mountain bighorn sheep (*Ovis canadensis canadensis*) may play a role in infection clearance (tumors are associated with pathogenic bacteria presence in the upper respiratory tract of bighorn sheep; Fox et al., 2015); however, the causative agent of tumors is unknown (Fox et al., 2016).

The outcome of *M. ovipneumoniae* infection in bighorn sheep—whether mortality, recovery, or transition to chronic carrier—is likely influenced by a multitude of factors, including pathogen strain, host immunity, host demographics (specifically, age, see Plowright et al., 2017), dosage, and environmental conditions. Here, we explore the association of host‐genomic variation and chronic carrier phenotype in a well‐studied bighorn sheep herd (Lostine herd, Hells Canyon) using family‐based genome‐wide association methodology and genome‐wide diversity metrics. Long‐term monitoring of the Lostine herd has identified chronic carriers, intermittent carriers, and noncarriers within the herd, and we hypothesize that host‐genomic variation plays a role in carrier phenotype. A previous study of the Lostine herd found a strong association between carrier status and heterozygosity at a major histocompatibility complex (MHC) locus, suggesting genetic diversity at the MHC gene complex may influence carrier status (Plowright et al., 2017). This prior study examined relationships between carrier status and diversity at four microsatellite markers associated with four genes with immune system functions, as well as eleven microsatellite markers in “neutral” genomic regions (i.e., not associated with genes); here, we expand on that study by investigating associations between carrier status and allelic composition at thousands of SNPs across the genome. We also estimate genome‐wide and MHC region diversity (heterozygosity and allelic richness) between phenotypes. Pathogen exposure, strain, and habitat conditions are similar across individuals within the Lostine herd, and therefore, this study system provides an opportunity to investigate genome‐wide associations without the confounding influence of these variables. Due to the difficulty in collecting longitudinal, invasive data in free‐ranging wildlife, our sample sizes are necessarily small; however, individuals within the Lostine herd have shared ancestry, and thus, we employ a family‐based GWA approach. This study represents one of the first to explore associations between genomic variation and carrier status in a free‐ranging species. Furthermore, our study demonstrates the potential power of family‐based genome‐wide association analyses to aid in management decision‐making for bighorn sheep and other wildlife species threatened by disease.

## 2 METHODS

### 2.1 Study population: Lostine herd

**FIGURE 1:**
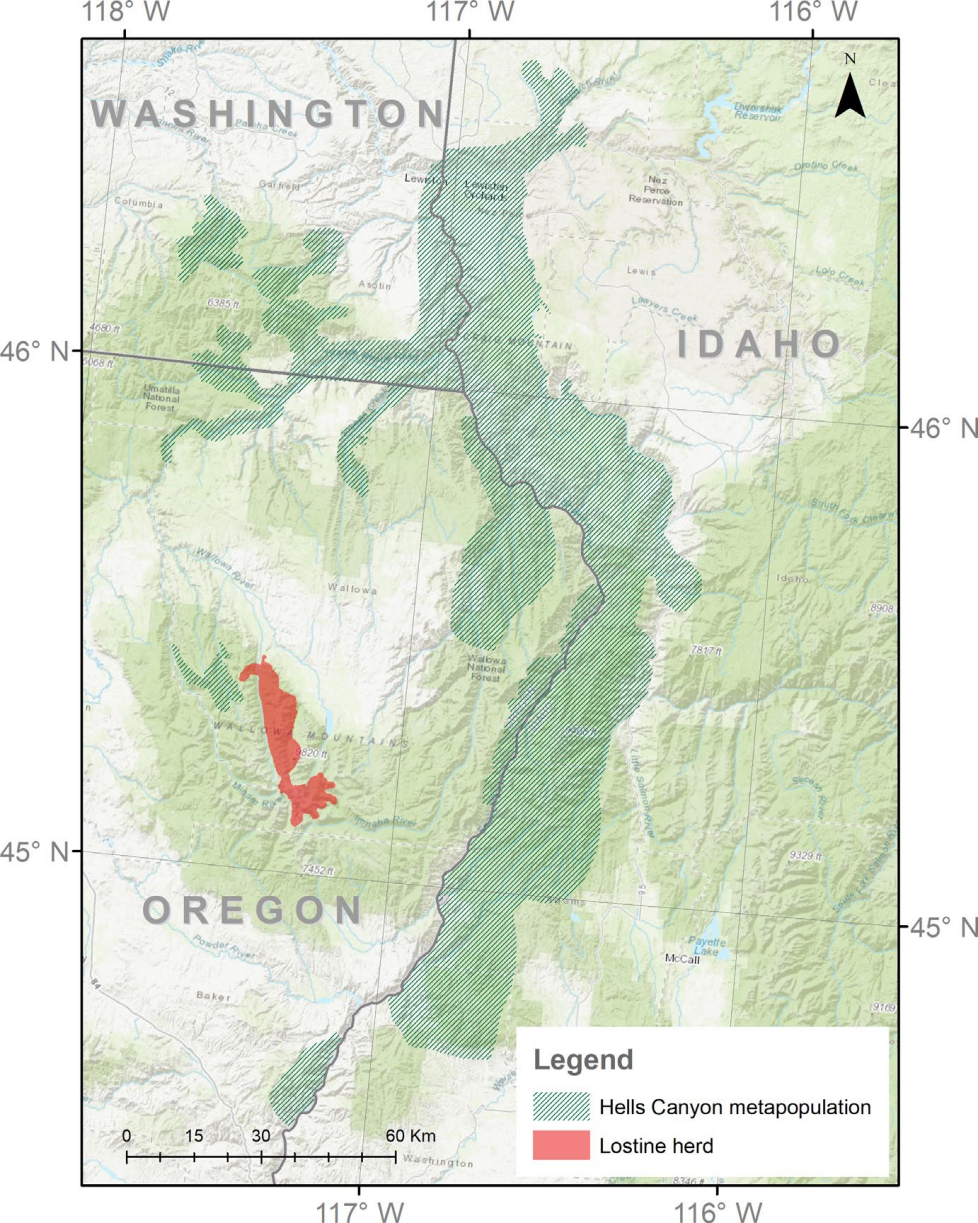
Distribution of the Hells Canyon bighorn sheep metapopulation across Idaho, Washington, and Oregon. This metapopulation consists of 18 herds including the Lostine herd (red). Herd range geospatial data were freely available from state wildlife agencies’ websites. The base map was sourced from ESRI (Esri & Nov., 2020)

The Lostine River bighorn sheep herd occupies an approximately 230 km^2^ range in the Wallowa Mountains of northeastern Oregon and is part of the Hells Canyon metapopulation, which includes 18 sheep populations and spans the states of Idaho, Oregon, and Washington (~2.3 million hectares, Figure 1; Fortin & Cassirer, 2015). Bighorn sheep are native to this region but were locally extirpated by 1945. The Lostine herd was re‐established through the translocation of 20 sheep from Alberta, Canada in 1971. Since the reintroduction event in 1971, the population has been monitored annually. Following population growth post‐introduction, management efforts were adopted to maintain the population at approximately 80 individuals through translocations of sheep out of the Lostine herd (Coggins, 2006). In 1986–87, a pneumonia outbreak resulted in the loss of two‐thirds of the population (Cassirer et al., 1996; Coggins, 1988). By 1997, the population had made a near‐full recovery reaching 92% of its pre‐epidemic size. In the early 2000s, a different *M. ovipneumoniae* strain that was associated with pneumonia epizootics throughout the Hells Canyon metapopulation was detected in the Lostine population (Cassirer et al., 2016), and since then, the population has been stagnant to declining, estimated at 60 – 90 sheep with no translocations out of the population (Oregon Dept. of Fish and Wildlife, unpublished data).

The Lostine herd is a well‐mixed population—with many related individuals—that winters on a range where the herd is supplementally fed as part of management efforts and where our genetic samples were collected. Disease exposure was homogeneous throughout the population as was confirmed by *M. ovipneumoniae* antibody presence in all sheep included in this study (Plowright et al., 2017). This is an important advantage in our association analysis, as results can be confounded by individuals that are phenotypically disease‐free due to lack of exposure, but exhibit “case” genotypes (i.e., individuals that have carrier genotypes but are classified as controls due to not being exposed). Further, a single *M. ovipneumoniae* strain was detected during the sampling period in the Lostine population, eliminating strain variation complexities (the same strain has been present since at least 2007; Cassirer et al., 2016).

### 2.2 Sample collection and infection status

From 2011 to 2016, sheep (lambs, yearlings, and adults) in the Lostine herd were baited with alfalfa pellets and captured in a corral trap or chemically immobilized using a jab stick or dart gun. Individuals were aged and given a unique marker for recapture, and samples were collected including skin tissue, blood, and nasal swabs (Plowright et al., 2017). A subset (*n* = 52) of individuals was periodically recaptured, allowing for assessment of longitudinal infection status. The sampling regime focused on ewes, given the research interests of the study for which these data were collected (Plowright et al., 2017). *Mycoplasma ovipneumoniae* infection status was determined using conventional and real‐time PCR on nasal swabs (see Plowright et al., 2017). Age was documented for each sheep at every capture event and is reported as a range spanning age at first capture to age at last capture (Table 1). A subset of individuals that died was necropsied postmortem, and sinus tumor presence was documented.

**TABLE 1:** Sheep infection status and sinus tumor presence. Sinus tumor presence is designated by a “+” symbol. Sinus tumor data were only available for five individuals

Sheep ID	Age range of testing (total tests)	Infection status	Sinus tumor presence
04LO58 (ARH1)	4.7–12.7 (7)	carrier	+
14LO36 (ARI1)	4.4–8.7 (3)	intermittent	no data
04LO73 (ARJ1)	2.7–15.7 (9)	intermittent	no data
14LO83 (ARR1)	2.8–4.8 (4)	carrier	+
14LO81 (ARS1)	2.8–7.7 (3)	intermittent	no data
13LO01 (ART1)	4.5–6.8 (6)	intermittent	no data
11LO44 (ARW1ab)	4.7–9.8 (7)	negative	no data
14LO91 (ARX1)	3.7–6.8 (2)	intermittent	no data
13LO82 (ARY1ab)	1.9–4.8 (3)	intermittent	no data
08LO24 (ARZ1)	12.7–17.7 (5)	intermittent	no data
12LO38 (AS81)	4.6–7.8 (6)	carrier	+
14LO05 (AS91)	4.4–5.8 (2)	intermittent	no data
14LO51 (ASA1)	4.6–6.8 (2)	negative	no data
02LO43 (ASB1)	13.9–15.7 (4)	carrier	no data
05LO53 (ASH1)	8.8–11.8 (5)	negative	no data
04LO74 (ASI1)	12.7–15.8 (5)	carrier	+
03LO50 (ASL1)	12.8–15.8 (5)	intermittent	no data
10LO33 (ASM1)	3.8–9.8 (7)	intermittent	+
08LO29 (ASN1)	4.8–8.7 (7)	intermittent	no data
11LO40 (ASQ1)	4.7–9.8 (6)	negative	no data
12LO56 (AST1)	4.6–6.8 (7)	intermittent	no data
13LO02 (ASV1)	4.6–6.8 (4)	negative	no data
99L09 (ASW1)	12.8–16.7 (5)	carrier	no data
15LO54 (BQS1ab)	0.7–4.7 (3)	negative	no data
15LO63 (BQU1ab)	1.7–5.6 (3)	negative	no data

Infection phenotype was assigned based on nasal swab PCR results with the following classifications: (i) individuals that consistently tested negative for *M. ovipneumoniae* through time were given the status “negative”; (ii) individuals that tested positive and negative in consecutive captures, that is, infected and recovered, were given the status “intermittent”; and (iii) individuals that tested positive in two consecutive sampling events across two or more years were given the status of “chronic carrier” (Table 1) (Plowright et al., 2017). Individuals had to be tested ≥2 times for inclusion in this study (see Table 1). Our main objective was to understand drivers for chronic (i.e., persistent) carrier status; thus, we combined the negative and intermittent classes into one “negative” control class, as this grouping allowed us to specifically address carriage.

### 2.3 DNA extraction and RADseq library preparation

Restriction site‐associated DNA sequencing (RADseq) data were available from a previous parentage study of the Lostine herd, including 52 individuals with longitudinal disease data (Andrews et al., 2018; NCBI BioProject ID PRJNA454718, SRA accession SRP144608). RADseq generates sequence data from loci distributed relatively randomly across the genome, enabling a survey of a large portion of the genome (Andrews et al., 2016). Samples used to generate RADseq data consisted of blood and skin tissue samples collected as described above. DNA extraction and RADseq library prep are described in Andrews et al. (2018). Briefly, DNA was extracted using the DNeasy Blood and Tissue Kit (Qiagen Inc., Germantown, MD, USA). RADseq libraries were constructed using 50 ng of high‐molecular‐weight genomic DNA. DNA was digested using the *Sbfl* restriction enzyme, and cut sites were ligated with biotinylated RADseq adapters containing 8 bp barcodes unique to each sample. Ligated products from all samples were multiplexed and sheared to 400 bp using a Covaris M220 Focused‐ultrasonicator. Streptavidin bead washes were used to remove DNA fragments that did not have ligated adapters, and remaining fragments were prepped for sequencing using NEBNext Ultra DNA Library Prep Kit for Illumina, followed by sequencing with an Illumina HiSeq4000 at the University of California Berkeley QB3 Vincent J. Coates Genomics Sequencing Library with 150 bp paired‐end reads.

### 2.4 SNP discovery and genotyping

Sequence reads containing Illumina adapters were identified and removed using HTS_ADATPERTRIMMER (https://github.com/s4hts/HTStream). Remaining reads were demultiplexed and cleaned using PROCESS_RADTAGS in STACKS v2.2 (J. Catchen et al., 2013) with the options ‐c, ‐q, ‐r, and ‐‐bestrad. PCR duplicates were removed using CLONE_FILTER in STACKS. Sequence reads were then merged for 18 samples for which library prep and sequencing had been performed twice independently (see Andrews et al., 2018).

Quality filtered bighorn sheep sequences were aligned to the domestic sheep (*Ovis aries*) genome (Oar v4.0) using end‐to‐end alignment methods in Bowtie2 v2.3.5.1 (Langmead & Salzberg, 2012). Parameters for alignment included the following: ‐sensitive (a default set of parameters for alignment sensitivity and accuracy) ‐X 900 (maximum fragment length for valid alignments). Reads were filtered with the program SAMtools v1.9 (Li et al., 2009) for mapping quality >40. Variant discovery and genotyping were performed in STACKS v2.4 (J. Catchen et al., 2013; J. M. Catchen et al., 2011), and loci were filtered by minimum depth (≥5) and minimum quality score (≥20). Loci were further filtered in PLINK v2.0 (Purcell et al., 2007) based on level of missing data per site (≤10%) and minor allele frequency (≥1%), followed by sample‐level filtering, which removed samples with >10% missing data. Linkage disequilibrium filtering was performed in PLINK v2.0 to retain a set of loci no less than 5 kb of each other (‐‐indep‐pairwise 5 5 0.2). This window is smaller than linkage decay observed in this population (~500 kbp; Andrews et al., 2018), but was employed to limit SNPs in close proximity while retaining the majority of loci. Lastly, loci were removed that departed from Hardy–Weinberg equilibrium (HWE); this was determined in PLINK v1.9 using only samples with the control phenotype, as to not filter out loci that may be associated with disease status (following suggestions by Reed et al., 2015). Population structure was tested using both the ‐‐cluster and ‐‐pca flags in PLINK (versions 1.9 and 2.0, respectively), and no differentiated groups were observed. To correct for the bimodal relationship between age and infection status—the proportion of individuals that test positive for *M. ovipneumoniae* is higher in young (≤3) and old (≥14) individuals (Plowright et al., 2017)—we removed individuals if their capture events occurred exclusively before the age of four or after the age of fourteen. Following filtering, 25 samples remained, all of which were female bighorn sheep. These filtering steps are summarized in Data S1.

### 2.5 Observed heterozygosity and allelic diversity

Several genomic diversity metrics were estimated to investigate the relationship between diversity and carrier status. Standard heterozygosity (proportion of heterozygous loci divided by the mean expected heterozygosity of typed loci) was calculated for each individual using the *genhet* function in R v3.5.3 (Coulon, 2010; R Development Core Team, 2016). The standard heterozygosity estimate accounts for differences in the numbers of genotyped loci among individuals. In addition, several population‐level diversity metrics were calculated to compare carrier and control phenotypes. Observed heterozygosity was estimated for each phenotype in R v3.5.3 using the *basic.stats* function in package *hierfstat* v0.5–7 (which accounts for group sample size; Goudet & Jombart, 2015). We also employed a rarefaction approach to estimate metrics of overall allelic richness (the average number of alleles per locus) and private allelic richness (number of unique alleles in a population) for both carrier and control phenotypes using program ADZE v1.0 (Szpiech et al., 2008). This rarefaction approach allows for comparisons among groups where sample sizes differ, as is the case for our carrier and control groups. The maximum standardized sample size was set to 6, which is the sample size of the carrier group. For each diversity metric, we used Welch's two‐sample *t* test in program R v3.5.3 to assess if mean diversity differed between control and carrier groups across all loci. We also implemented each of these diversity analyses to compare heterozygosity, allelic richness, and private allelic richness between phenotypes for loci that fell within the *Ovar‐Mhc* region—where the major histocompatibility complex (MHC) class I through III genes are located—on chromosome 20. *Ovar‐Mhc* SNPs were identified from base 7,166,117 to 27,665,927 spanning the locations of the DYA to OLA genes based on our current understanding of the *Ovar‐Mhc* region structure and location in domestic sheep (Dukkipati et al., 2006).

### 2.6 Association test

A single‐marker univariate linear mixed model was used to test for SNP associations with pneumonia carrier status. Phenotypes were divided into one binary trait: *M. ovipneumoniae* negative (controls; individuals testing consistently negative or intermittently positive for *Mycoplasma*; *n* = 19) and *M. ovipneumoniae* carriers (cases; always *Mycoplasma* positive, *n* = 6) and analyzed using the following model (Zhou & Stephens, 2012, 2014):

(1) y=Wα+xβ+u+ϵ

where y is a vector of binary disease labels (0 for negative, 1 for carrier), W is a matrix of covariates including a column of ones (no additional variables included in this analysis), α is a vector of corresponding coefficients (including intercept), x is a vector of SNP genotypes, β is the corresponding effect of SNPs, u is a vector of random effects, and ϵ is a vector of errors. The random effect term (u) can incorporate a relatedness matrix (centered relatedness matrix generated in GEMMA; Data S2), which we included to account for relatedness as we observed many closer‐than third‐degree relatives in this population (see also the kinship coefficient matrix from PLINK v2.0; Data S2). The relatedness matrix was estimated using all sheep with RADseq data in the Lostine population (*n* = 82; Andrews et al., 2018), not just the individuals with longitudinal disease information used in the GWA. The association analysis was performed using GEMMA v0.98.1 (using ‐miss 0.1 ‐hwe 0.000001 ‐maf 0.01‐km 1 ‐lmm 4; Zhou & Stephens, 2012), and the likelihood ratio test *p‐*values were used to test the null hypothesis (β = 0) for each SNP. The *p‐*value significance threshold was adjusted using Bonferroni correction resulting in a threshold of 4.71 × 10^–6^. Further, as the Bonferroni method can be overly conservative, we controlled for type I errors by using false discovery rate (FDR) methodology, whereby new significance values (q‐values) and threshold are defined: in this case, we use α = 0.15 (15% of significant values are expected to be false positives) (Benjamini & Hochberg, 1995). Manhattan and quantile–quantile plots (Q‐Q plot) were created using the *qqman* package v0.1.4 in R (Turner, 2014).

### 2.7 Identification of candidate genes

Genes with potential for influencing carrier status were identified as any genes located within a 500 kb window (up‐ or down‐ stream) of SNPs associated with persistent carriage. This window is based on linkage decay reported for this RADseq dataset (Andrews et al., 2018). Candidate genes were visualized using the NCBI Genome Data Viewer (https://www.ncbi.nlm.nih.gov/genome/gdv) and the domestic sheep genome (*Ovis aries,* Oar v4.0; GenBank accession GCA_000298735.2; annotation release 102).

### 2.8 Gene ontology enrichment analysis

We implemented an overrepresentation test using PANTHER (http://geneontology.org/; Mi et al., 2018), which identifies over‐ or under‐represented gene ontology terms for a gene subset in relation to a reference gene set. The subset of genes associated with the significant SNPs identified by the family‐based GWA analysis (the candidate genes identified by the previous section) was compared to a reference gene set comprised of all genes within a 500 kb window of all SNPs used in the analysis. To determine the list of genes in the reference set, we first used the UCSC Genome Table Browser database to obtain a full list of annotated genes with their positions on the *O. aries* v4.0 genome (https://genome.ucsc.edu/cgi‐bin/hgTables; Karolchik et al., 2004). We then used BEDTools v2.29 (Quinlan & Hall, 2010) to identify genes positioned within a 500 kb window of all loci used in the family‐based GWA. The PANTHER analysis utilized biological processes, cellular components, and molecular functions identified for genes in the human genome and applied Fisher's exact test, and *p*‐values were corrected using false discovery rate (FDR) calculations.

### 2.9 Genetic architecture

To understand if pneumonia carrier status is polygenic (many loci influencing the phenotype, each having little effect) or oligogenic (few loci affecting the phenotype, each having a large effect), a Bayesian sparse linear mixed model (BSLMM) was implemented in GEMMA v0.98.1 (Zhou et al., 2013). The structure of the model was as follows:

(2) $$y=1_{n}\mu +x\beta +u+\epsilon$$

where 1*_n_* is *n*‐vectors of 1s, *μ* is a scalar for phenotype mean, x is a vector of SNP genotypes, β is the corresponding genetic marker effects, u is a vector of random effects (relatedness matrix), and ϵ is a vector of errors. The BSLMM estimates the proportion of variance in phenotypes explained by the SNPs using x*β* and u (PVE; “chip heritability”) and estimates a parameter rho whereby values close to 0 imply a polygenic architecture and values close to 1 imply oligogenic structure (Zhou, 2016). The probit BSLMM was run for 50 million sampling iterations following a 500k iteration burn‐in, and parameter estimates were recorded every 10 iterations for a total of 500,000 sampled values. While this method can be limited by small sample size, we report the results and acknowledge a level of uncertainty.

## 3 RESULTS

### 3.1 Sample size and diversity metrics

A total of 98,307 variant loci were identified using genomic data from 52 bighorn sheep (19 carriers and 33 control genotypes). After quality filtering of the sequence data (see Data S1), a total of 10,605 SNPs (of which *n* = 39 aligned to unplaced scaffolds) were identified and included in the genome‐wide association analysis. Of these, *n* = 151 SNPs occurred within the MHC gene complex, including *n* = 124 SNPs in the MHC class II region and *n* = 27 SNPs in the MHC class III region (no SNPs occurred in the MHC class I region). Details regarding final SNP locations and coverage across chromosomes can be found in Data S3. After controlling for age, a total of 25 individuals (all female) remained, with 19 having control (noncarrier) status and six having carrier status (Table 1). Mean individual standardized heterozygosity did not differ between carrier and control groups (mean_carrier_ = 1.03, mean_control_ = 1.04, *t*=−0.32, *df* = 6.03, *p* = .76). Further, there was no difference in observed population‐level heterozygosity between carrier and control groups for the 10,605 loci (mean_carrier_ = 0.25, mean_control_ = 0.25, *t*=−1.09, *df* = 20,321, *p* = .28), or for loci that fell within the *Ovar‐Mhc* region (mean_carrier_ = 0.23, mean_control_ = 0.24, *t* = −0.45, *df* = 293.13, *p* = .65). The mean allelic richness across all 10,605 loci was 1.55 (variance 0.14) and 1.55 (variance 0.10) for carrier and control groups, respectively (*t* = −0.10, *df* = 20,482, *p* = .92), and mean private allelic richness was 0.17 (variance 0.03; *t* = −0.19, *df* = 21,087, *p* = .85) for both groups when rarefaction subsample size was set to 6. Similarly, no difference in allelic richness was observed across loci within the *Ovar‐Mhc* region: mean allelic richness across was 1.51 (0.15 variance) and 1.51 (variance 0.12) for carrier and controls (*t* = −0.04, *df* = 296.53, *p* = .97), and mean private allelic richness was 0.16 (variance 0.03 and 0.04 for carriers and controls, respectively; *t* = −0.07, *df* = 295.94, *p* = .94).

### 3.2 Single SNP association

**FIGURE 2:**
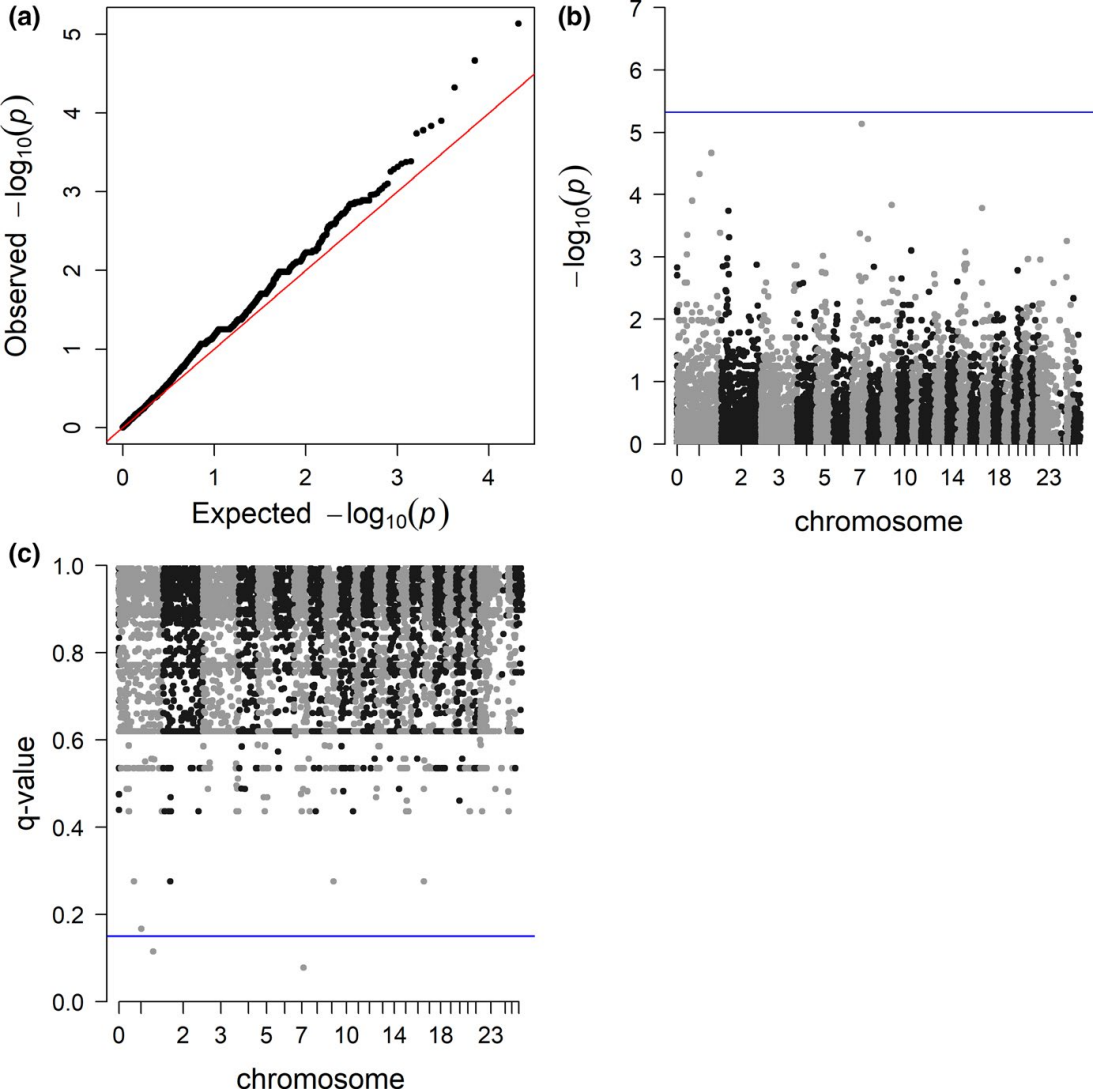
Q‐Q (a), Manhattan (b), and false discovery rate (c) plots for the results of the family‐based genome‐wide association of the *M. ovipneumoniae* chronic carrier trait. (b) The Manhattan plot displays the chromosomal location and ‐log_10_(*P*‐value) for each variant included in the association test (represented as points). The Bonferroni‐corrected threshold of 4.71 × 10^–6^ (5.32 on ‐log_10_ scale) is designated by the blue horizontal line. (c) False discovery rate corrected q‐values are plotted with a cutoff of 0.15 (blue horizontal line). Two SNPs are identified as being significant (see Table 2)

**FIGURE 3:**
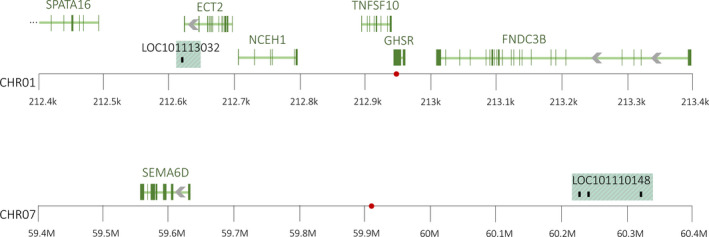
Genome view of phenotype associated single nucleotide polymorphism (SNPs) and peripheral protein‐coding regions. Locations of SNPs identified as associated by the analysis are shown in red. Gene regions are defined by green horizontal bars (intron regions) and green vertical bars (feature intervals or exons). Gray left‐facing arrows indicate that the region is located on the complement. Pseudogenes are designated by the green‐striped shading and exons in these regions are represented by vertical black bands

The Manhattan and Q‐Q plots for the carrier trait association analysis are shown in Figure 2. After implementing false discovery rate methods, the univariate linear mixed model identified two SNPs that were associated with the carrier phenotype (Figure 2c). The first SNP (ID: 14,407:36:+, Table 2) fell within the 5’ untranslated mRNA region of the growth hormone secretagogue receptor gene (*GHSR*) on chromosome 1. Further, within the 500 kb up‐ and down‐stream window of this SNP, there were five genes of interest (Table 2, Figure 3). The second associated SNP was positioned on chromosome 7 of the *O. aries* genome in a noncoding region, with one additional gene of interest within the 500 kb window (Table 2). The functions of these potential candidate genes are outlined in Table 3 and were determined using the National Center for Biotechnology Information and UniProt databases, https://www.ncbi.nlm.nih.gov/gene and https://www.uniprot.org, respectively (Hutchins, 2014), as well as published literature. Additional information regarding candidate gene function, processes, and disease associations are outlined in Data S4.

**TABLE 2:** Single nucleotide polymorphism (SNP) loci identified as significant in the family‐based genome‐wide association analysis (*n* = 2). Allele frequencies were calculated using PLINK v1.9 (‐‐assoc command)

Chr	ID / position	region	*P*‐val	q‐val	A1	A2	f(A)	f(U)	H_e_	LD region
1	14,407:36:+ 212,949,890	gene region, mRNA UTR	2.2E−05	0.115	T	C	0.00	0.47	0.56	Gene(s): ***GHSR***, SPATA16, ECT2, NCEH1, TNFSF10, FNDC3B Pseudogene(s): LOC101113032
7	182,910:31:‐ 59,906,180	noncoding	7.3E−06	0.078	G	A	0.67	0.13	0.28	Gene(s): SEMA6D Pseudogene(s): LOC101110148

Note

Chr, chromosome; ID, variant identification; UTR, untranslated region; A1, allele 1 (minor allele); A2, allele 2 (major allele); f(A), frequency of allele 1 among individuals with the case phenotype (chronic carrier); f(U), frequency of allele 1 observed in individuals with the control phenotype (noncarrier); H_e_, observed heterozygosity at locus; LD region, genes, pseudogenes, or protein‐coding regions within ± 500 k base pairs up‐ or down‐stream of the SNP location (within the linkage disequilibrium decay threshold).

**TABLE 3:** Description of candidate gene function and tissues where they are expressed in *O. aries*. Information regarding the tissues where the highest expression of each gene is observed in *O. aries* was obtained from the National Center for Biotechnology Information website (https://www.ncbi.nlm.nih.gov/gene/). Additional information regarding biological processes for each gene can be found in Data S4

Gene	Summary of gene function	Highest expression in *O. aries*	Citation
GHSR: growth hormone secretagogue receptor	(1) Stimulation and release of hormones, (2) regulation of energy metabolism and food intake, (3) regulation of cell growth and survival, (4) pancreatic function, (5) regulation of immune functions related to aging and gastrointestinal homeostasis, (6) mitigation of inflammatory processes, and (7) cardio vascular and nervous system cell protection. Can be expressed in select forms of cancer	spleen, prescapular and mesenteric lymph node, omentum, lung tissues	Taub, 2007; Yin et al., 2014
SPATA16: spermatogenesis associated 16	Influences spermatozoa production	testes	
ECT2: epithelial cell transforming 2	Role in Rho activation (an essential protein that functions in furrow formation during cell cleavage. Overexpression of *ECT2* has been observed in tumor tissues in humans. Also involved in enzyme regulator activity and enzyme binding	placental tissues, whole embryos	Yüce et al., 2005; Sano et al., 2006
NCEH1: neutral cholesterol ester hydrolase 1 (also called KIAA1363)	Plays role in hydrolysis of intracellular cholesterol ester and ether lipid signaling network. Elevation of *NCEH1* has been documented in cancer cells; suppression of *NCEH1* expression inhibits tumor cell migration and growth	cerebrum, hypothalamus, brain stem, alveolar macrophages	Chiang et al., 2006; Igarashi et al., 2010
TNFSF10: TNF superfamily member 10	Induces apoptosis in neoplastic cells (abnormal cell masses, such as tumors), plays role in suppressing cancer cells and their metastases	lymph node, lung tissue	Griffith et al., 1998; Hellwig & Rehm, 2012
FNDC3B: fibronectin type III domain containing 3B	Potential to act as an oncogene when overexpressed, may promote tumor growth	placental tissues	Cai et al., 2012; Lin et al., 2016
SEMA6D: semaphorin 6D	(1) Immune function, (2) osteoclastogenesis, (3) cardio morphogenesis. Putative role in cancer	adrenal gland, brain stem, mammary gland, corpus luteum, hypothalamus, lung tissues	Kumanogoh & Kikutani, 2010; Kang & Kumanogoh, 2013; Chen et al., 2015; Moriarity et al., 2015; Lu et al., 2016

### 3.3 Enrichment analysis

The gene ontology biological process enrichment analysis compared seven candidate genes to 1,003 reference genes identified by the USCS table browser to be ± 500 kb from any SNPs in the dataset. The reference genes related to 8,884 biological processes (GO terms), 1,709 molecular functions, and 879 cellular mechanisms in the human genome. After FDR corrections, no gene ontology terms were found to be overrepresented in any of the three categories.

### 3.4 Genetic architecture

The BSLMM revealed that 10,605 SNPs explain approximately 55.9% (PVE median, mean 54.5% ±32 S.D.) of the phenotypic variance, with a select few (median *n* = 16 SNPs) having a relatively large effect, explaining 45.2% (PGE median, mean 46.2% ± 31.7 S.D.) of phenotypic variance (Table 4). The value for rho (0.50, median) does not clearly identify the genetic architecture as either polygenic or oligogenic (Table 4). These results yield high uncertainty (see Table 4 and sampling distributions in Data S5), which is likely the result of small phenotypic sample size. While convergence was difficult to attain for some parameters (n[gamma] and pi), conservative inference may be made for others (PVE, PGE, and rho).

**TABLE 4:** Results from the Bayesian sparse linear mixed model assessing heritability of carriage. There was considerable uncertainty in n.gamma estimates (see Data S5) that could not be resolved

Parameter	Mean (*SD*)	Median
Variance explained by all genotypes (PVE)	0.545 (0.320)	0.559
Number of large‐effect SNPs (*n*.gamma)	82.55 (168.11)	16
Variance explained by large‐effect SNPs (PGE)	0.462 (0.317)	0.452
Genetic architecture (rho)	0.506 (0.288)	0.509

Note

PVE, phenotypic variance explained by all SNPs; PGE, phenotypic variance explained by large‐effect SNPs (n.gamma); n.gamma, number of large‐effect SNPs; rho, value for genetic architecture; SD, standard deviation. Means and medians were calculated from the posterior distribution.

## 4 DISCUSSION

Pneumonia resulting from *M. ovipneumoniae* infection is one of the major factors impeding the recovery of bighorn sheep in North America. Outbreaks can result in devastating all‐age die‐offs, followed by limited lamb recruitment due to chronic carriers remaining in affected populations (Cassirer et al., 2018; Garwood et al., 2020). Here, we explored the impact of host genomics on the postoutbreak chronic carrier disease phenotype in bighorn sheep. We analyzed 10,605 single nucleotide polymorphisms from across the bighorn sheep genome and reveal two loci associated with the chronic carrier phenotype. Further, a total of seven candidate genes were identified as potentially influencing disease phenotype. No differences were observed in heterozygosity or allelic richness between phenotypes. While conclusions here are limited by sample size, these findings present a first step toward understanding the genomic basis of chronic carriage in bighorn sheep and lay the foundation for future research. Larger sample sizes for both carriers and controls (of both sexes) as well as the inclusion of sheep from additional herds are needed to further explore the genomic associations identified here.

### 4.1 Genetic diversity and *M. ovipneumoniae* carriage

Loss of genetic diversity and inbreeding are expected to leave host populations vulnerable to disease risk (Spielman et al., 2004). Further, polymorphisms near‐to or within genes involved in immune function may play an important role in an individual's susceptibility, resistance, and tolerance to disease (O'Brien & Evermann, 1988). Here, we compared heterozygosity and allelic richness, across the entire SNP panel and for SNPs located within the MHC region, for *M. ovipneumoniae* carrier and control groups in bighorn sheep.

Host heterozygosity has often been associated with genetic health and the ability to resist pathogen infection (e.g., heterozygous advantage; Spurgin & Richardson, 2010). This idea has been previously demonstrated in free‐living sheep. For example, inbred Soay sheep (*Ovis aries*)—as assessed by heterozygosity at several microsatellite loci—were found to be more susceptible to gastrointestinal parasites (Coltman et al., 1999). In the bighorn sheep population studied here, differences in heterozygosity between *M. ovipneumoniae* carriers and noncarriers were previously assessed at eleven neutral and four putatively adaptive microsatellite loci (Plowright et al., 2017). Plowright et al. (2017) found that one of the four putatively adaptive loci (located in the MHC I gene complex) had significantly lower heterozygosity in persistent carriers relative to noncarriers; the other three loci, which occurred in other immune system‐related genes, showed no differences in heterozygosity. Plowright et al. (2017) also found no differences in heterozygosity between groups at the eleven neutral microsatellite loci. Similarly, we detected no difference in population‐ or individual‐level heterozygosity across our full set of SNP loci between carriers and noncarriers. We also found no difference in heterozygosity between groups when comparing loci located in the *Ovar‐Mhc* region on chromosome 20. Notably, however, all of these loci occurred within the MHC II or MHC III complexes, and none occurred within the MHC I gene complex, where the microsatellite locus in Plowright et al. (2017) occurred. Though heterozygosity has been associated with host susceptibility and resistance in some wildlife disease systems, this may vary depending on the host and pathogen. Here, we found no association between heterozygosity and pneumonia carriage in bighorn sheep.

Allelic richness—or, the average number of alleles per locus—is another metric by which genetic diversity can be assessed and is a reliable predictor of a population's potential adaptability (Caballero & García‐Dorado, 2013). A related metric, private allelic richness (the number of unique alleles in a group) is used to genetically distinguish groups from one another (Kalinowski, 2004). We assessed allelic richness in our dataset using rarefied methods to account for disparities in sample sizes and found no difference in overall allelic richness or private allelic richness between phenotypes. The lack of significant differences for genetic diversity observed between phenotypes for all diversity measures suggests that genome‐wide diversity is likely not predictive of carrier status.

### 4.2 Candidate genes and sinus tumors

Seven candidate genes were identified nearby to the SNPs associated with chronic carrier status, using the annotation of the domestic sheep (*O. aries*) genome (Oar v4.0). Of these seven, six genes—*ECT2*, *NCEH1*, *TNFSF10*, *FNDC3B, GHSR,* and *SEMA6D*—have either been observed at elevated expressions in tumor cells or have been documented acting as oncogenes for specific cancers in humans (genes that can transform cells into tumor cells). Changes in expression of these genes may influence tumor regulation in the bighorn sheep host.

Paranasal sinus tumors in Rocky Mountain bighorn sheep are characterized by thickening of the sinus lining (maxillary and/or frontal sinuses) and neoplasm exudate (liquid secretion from abnormal growths or damaged tissues) (Fox et al., 2010). Sinus tumors were a good predictor of pathogenic bacteria (including *M. ovipneumoniae*) carriage in the upper respiratory tracts of bighorn sheep in a Colorado population (Fox et al., 2015). Specifically, 36% of sheep with sinus lining thickened >5 mm were PCR positive for *M. ovipneumoniae* (as opposed to 13% PCR positive with no tumor presence). These findings suggest that sinus tumors may disrupt the host's ability to clear pathogens, allowing for maintenance of chronic infections in the upper respiratory tract and potential prolonged shedding (Fox et al., 2015). In our study, six of the seven candidate genes associated with persistent pathogen carriage have functions in tumor regulation, potentially indicating that a genetic predisposition to tumor growth influences pathogen carriage. However, no gene ontology terms were found to be significantly overrepresented for these seven candidate genes. Additional research would help assess the potential for a genetic predisposition for tumor development, and consequently, if this impacts the risk of chronic carriage of *M. ovipneumoniae*.

In our dataset, information regarding the presence or absence of sinus tumors was available for five individuals (four chronic carriers and one intermittent carrier), and each of these individuals had sinus tumors (Table 1). The presence of tumors in all four chronic carriers supports the hypothesis that tumors prevent pathogen clearing and promote long‐term pathogen shedding (high infectivity, superspreaders). However, tumor data were not available for the remaining samples, thus limiting the conclusions that can be drawn regarding the association between carrier status and tumor presence for this dataset. Additional monitoring for tumors in both control and carrier sheep is necessary to better understand the role of tumors in pneumonia carriage in bighorn sheep.

### 4.3 Mutation in the growth hormone secretagogue receptor (GHSR) gene

Two SNP variants were identified as being associated with *M. ovipneumoniae* carrier status in bighorn sheep. One variant was located in the 5’ untranslated region (5’UTR; mRNA leader sequence) of the growth hormone secretagogue receptor (*GHSR*) gene. The *GHSR* gene encodes a G‐protein‐coupled receptor that binds ghrelin, a peptide hormone, and is most widely recognized for its role in energy metabolism and growth (Müller et al., 2015). However, ghrelin and its receptor *GHSR* may play a complex role in the regulation of several other physiological functions, including immune function (Dixit & Taub, 2005; Yin et al., 2014). The variant identified by the genome‐wide association analysis falls within the 5’ untranslated region of the *GHSR*, a region that does not directly translate into proteins; however, both 5’ and 3’ untranslated regions are transcribed and function in post‐transcriptional regulation (Hubé & Francastel, 2018). In instances when a start codon is located in the 5’ untranslated region, peptide (short chain of amino acids) translation can occur, which may affect expression of down‐stream proteins (Calvo et al., 2009). However, these regions—known as upstream open reading frames—are not present in the 5’ untranslated region of the *GHSR* gene in the *O. aries* genome (assembly version 4.0).

In domestic bovids, mutations in the *GHSR* gene have been of interest for selective breeding potential and quality assessment. Polymorphisms identified in the 5’ untranslated region of the *GHSR* were associated with increased carcass weight and average daily gain in domestic cattle (Komatsu et al., 2011). In caprine, investigations of polymorphisms in the protein‐coding region of the *GHSR* gene have been associated with changes in body metrics, including body weight, body length, blood cholesterol, and abdominal fat in domestic sheep (Bahrami et al., 2012), and body weight and chest depth in domestic goats (Da et al., 2013). Past research has indicated that ghrelin and *GHSR* may play an important role in inflammation regulation (suppression; Dixit & Taub, 2005), and *GHSR* mRNA expression in human leukocytes (T and B cells and monocytes) suggests the ghrelin/*GHSR* pathway may have a role in generating or controlling immune response (Dixit & Taub, 2005; Taub, 2007). In the Lostine bighorn sheep herd, neither carcass trait data nor immune function data were available, limiting our understanding of the impact that the *GHSR* 5’ untranslated region mutation may have had on bighorn sheep body metrics and immune response.

### 4.4 Genetic architecture of carriage—polygenic?

Genetic architecture refers to the genetic contribution to—or heritability of—a given phenotype, specifically whether the phenotype is influenced by few or many variants and the respective contribution of each variant to the phenotype. For example, in wild bighorn sheep, narrow‐sense heritability of fitness‐related traits, specifically horn size, has been investigated using quantitative trait loci (QTL) (Johnston et al., 2010; Poissant et al., 2012). However, heredity of disease‐related traits—such as susceptibility, resistance, and infectivity—can be difficult to quantify in wildlife. An understanding of genetic architecture for disease phenotypes can provide important insight into effective mitigation practices, including risk assessment, drug development, and screening (Timpson et al., 2018). For example, in domestic sheep, heritability of susceptibility to ovine footrot was found to be ~40% and the genetic architecture was polygenic (many loci contributing a small amount to phenotype), revealing that genetic screening would not suffice for assessing sheep quality (Raadsma et al., 2018).

Here, we found that our SNP panel accounted for ~55% of the phenotypic variance (chip heritability) in the carriage phenotype, with large‐effect variants contributing to approximately 25% of the total phenotypic variance (or approximately 45% of the total variance explained by all SNPs). However, there is considerable uncertainty surrounding both estimates: the number of large‐effect SNPs could not be quantified with confidence, likely due to our relatively small sample sizes. Our results preliminarily suggest that more SNPs may have a large effect on the carrier phenotype than just the two identified in our analysis. However, our confidence intervals were broad, and additional studies with increased sample sizes would be required to fully characterize the genetic architecture of the carrier phenotype. A greater understanding of the numbers and effect sizes of genetic variants that influence resistance would allow managers to better assess the utility of using genomic screening moving forward. Specifically, the presence of few, large‐effect SNPs would allow for more focused carrier screening, as opposed to many, small effect variants spread across the genome.

### 4.5 Future directions

This study provides preliminary evidence of correlations between genotype and disease carriage in bighorn sheep. Due to the inherent difficulty in longitudinal sampling of wildlife, this study is not without limitations. While we employed conservative methods for assigning carriage status, there remains uncertainty and potential for error in carriage assignment. Discrepancies in the number and timing of *M. ovipneumoniae* tests performed per individual may have influenced carriage status. As there is a general U‐shaped trend for chronic carriage and age (Plowright et al., 2017), we attempted to control for this by eliminating individuals that were sampled at ages <4 or >14, exclusively. The limitations presented by testing number are more difficult to control for and are in‐part a function of field sampling opportunities. Additional genome‐wide association analyses in other bighorn populations are needed to test these associations and to provide greater insight into associations between genomic composition, tumor susceptibility, and pathogen carriage. Larger sample sizes—both in number of individual sheep and in number of tests per sheep—would increase statistical power for identifying genomic associations. Increasing testing per sheep across different ages would increase confidence in carriage assignment and reduce error in the independent variable. Further, increasing the number of genomic markers surveyed in these studies would also improve our understanding of these associations. The RADseq approach used here surveyed thousands of markers across the genome with the caveat that identified associated variants may not truly impact the observed phenotype, but may be linked with an unidentified polymorphism that influences phenotype. In contrast, a whole‐genome sequencing approach, while substantially more expensive, would provide a more thorough assessment of genomic associations and would have greater ability to identify variants that directly impact the phenotype (Visscher et al., 2017).

The association we identified here between persistent pathogen carriage and tumor regulation genes prompts additional questions that should be explored. First, to further understand the relationships between carriage and genomic variation related to tumor regulation in bighorn sheep, additional representative sampling is required from both carrier and noncarrier bighorn sheep of both sexes and across populations infected with other strains of *M. ovipneumoniae*. Additional approaches could include investigation of associations between carriage and a targeted subset of genes known to be associated with cancer, for example, using an amplicon sequencing approach (Meek & Larson, 2019). This approach would be substantially less expensive than RADseq or whole‐genome sequencing. Other approaches that would help elucidate the relationships between genomic composition, tumor susceptibility, and persistent carriage include (a) assessing the differences in candidate gene expression in chronic carriers relative to noncarriers, (b) exploring external factors (e.g., viruses) as potential instigators for tumor development, (c) documenting tumor prevalence in both disease classes (carriers and noncarriers) in additional populations where tumors are present, and iv) investigating the possibility that tumor development is instigated by infection with etiological agents of pneumonia, rather than persistent pneumonia infection being instigated by tumor development.

## 5 CONCLUSIONS

Here, we investigated the influence of genomic composition on persistent carriage of *M. ovipneumoniae* in bighorn sheep. To our knowledge, this is the first study to investigate the connection between genomic composition and persistent carriage in a free‐living species using restriction site‐associated DNA sequencing (RADseq) technology. We observed no difference in genome‐wide diversity (allelic richness and heterozygosity) between phenotypes. However, we did identify two variant loci and seven genes associated with carrier status, and six of these genes have functions related to tumor regulation. This result suggests potential causative links between genomic composition, tumor susceptibility, and pneumonia carriage. Support for this causative association comes from previous studies indicating a connection between paranasal sinus tumors and pneumonia in bighorn sheep. However, we observed no overrepresentation in our gene ontology analysis, and our interpretations are limited by our small sample size. This study provides a starting point for understanding the genomic basis and biological mechanisms underlying inability to clear infection in bighorn sheep, both of which are poorly understood but could provide important insight for understanding factors contributing to chronic pathogen carriage and potentially contributing to effective management strategies to combat pneumonia in bighorn sheep populations.

## CONFLICT OF INTEREST

The authors declare no competing interests.

## AUTHOR CONTRIBUTION

**Alynn Martin:** Conceptualization (equal); Formal analysis (equal); Methodology (equal); Writing‐original draft (equal); Writing‐review & editing (equal). **E. Frances Cassirer:** Conceptualization (equal); Data curation (equal); Funding acquisition (equal); Investigation (equal); Project administration (equal); Resources (equal); Writing‐original draft (equal); Writing‐review & editing (equal). **Lisette Waits:** Conceptualization (equal); Data curation (equal); Funding acquisition (equal); Project administration (equal); Resources (equal); Supervision (equal); Writing‐review & editing (equal). **Raina K Plowright:** Conceptualization (equal); Data curation (equal); Funding acquisition (equal); Investigation (equal); Writing‐review & editing (equal). **Paul Cross:** Conceptualization (equal); Resources (equal); Supervision (equal); Writing‐review & editing (equal). **Kimberly Andrews:** Conceptualization (equal); Data curation (equal); Formal analysis (equal); Funding acquisition (equal); Investigation (equal); Methodology (equal); Project administration (equal); Supervision (equal); Writing‐original draft (equal); Writing‐review & editing (equal).

## Supporting information

Supplementary Material

## Data Availability

The RADseq sequence data are available in the National Center for Biotechnology Information (NCBI) Short Read Archive: BioProject ID PRJNA454718 (https://www.ncbi.nlm.nih.gov/bioproject/), SRA accession SRP144608 (https://www.ncbi.nlm.nih.gov/sra/). Sequences are demultiplexed and quality filtered (using process_radtags in STACKS).
