# Supplementary material for: Genomic association with pathogen carriage in bighorn sheep (Ovis canadensis)
Source: Ecol Evol. 2021 Mar 2;11(6):2488–502. doi: 10.1002/ece3.7159 (PMC7981200; doi:10.1002/ece3.7159)
Supplement: Supplementary file 1 — Supplementary Material [file ECE3-11-2488-s001.docx]

**Supplemental Information for:**

**Genomic association with pathogen carriage in bighorn sheep (Ovis canadensis)**

Alynn M. Martin, E. Frances Cassirer, Lisette P. Waits, Raina K. Plowright, Paul C. Cross, Kimberly R. Andrews

**Table of Contents:**

| I. Loci filtering for genome wide association analysis. | Page 2 |
| --- | --- |
| II. Centered relatedness matrix produce by GEMMA for 25 bighorn sheep from the Lostine  population in eastern Oregon. | Page 3 |
| III. Final SNP coverage per chromosome of the *Ovis aries* genome (total SNPs = 10,605; of which 39 are on unplaced scaffolds). | Page 4 |
| IV. Biological processes, functions, and disease term associations for candidate genes. | Page 5 |
| V. Parameter value estimates from the Bayesian sparse linear mixed model (BSLMM). | Page 6 |
| References | Page 7 |

I. Loci filtering for genome wide association analysis. The final number of samples and loci used in the association analysis were 25 and 10,605, respectively.

| **Step** | **Details** | **N individuals** | **N loci** |
| --- | --- | --- | --- |
| Alignment | Align individuals with longitudinal disease data to reference genome | 52 | - |
| Filter loci | Alignment minimum quality score (≥20), and minimum depth (≥5) | 52 | 98,307 |
| Filter loci | Remove sites with >10% missing data and minor allele frequencies (1%) | 52 | 17,682 |
| Filter individuals | Remove samples with >10% missing data | 41 | 17,682 |
| Filter loci | Loci filtered based on linkage disequilibrium (within a 5k base pair window) | 41 | 11,890 |
| Filter loci | Use control phenotypes only to test if loci are out of Hardy Weinberg Equilibrium. Remove loci out of HWE | 41 | 11,890 |
| Filter individuals | Remove samples based on bi-modal age distribution whereby older and younger individuals are more likely to be infected with *M. ovipneumoniae* | 25 | 11,890 |
| GWAS | Gemma implements default filtering, including: removal of non-polymorphic sites, minor allele frequency (1%), and loci level missingness (10%). Further, final loci were filtered again by HWE (0.000001). | 25 | 10,605 |

II. Centered relatedness matrix produce by GEMMA and kinship relationship matrix produced by PLINK for 25 bighorn sheep from the Lostine population in eastern Oregon. The centered relatedness (bottom left) values are calculated using all sheep sampled in the population (n=82), even though only a subset is included in the analysis. The kinship coefficients (top right) from PLINK (v2.0; --make-king) were estimated using the final 25 individuals. Kinship coefficients for first-, second-, and third- degree relations are ~0.25, ~0.125, ~0.0625, respectively.

III. Final SNP coverage per chromosome of the *Ovis aries* genome (total SNPs = 10,605; of which 39 are on unplaced scaffolds). A rough estimate of chromosome coverage is calculated as the number of SNPs identified per chromosome divided by the quotient of total chromosome size and linkage block size (500 kb). Importantly, our SNP data are not distributed evenly across the genome, and thus what we report as ‘coverage’ is a merely a proxy for true coverage.

| **Chrom** | **Size** | **SNPs** | **Coverage (%)*** | **Chrom** | **Size** | **SNPs** | **Coverage (%)** |
| --- | --- | --- | --- | --- | --- | --- | --- |
| 1 | 275,406,953 | 1,091 | 1.981 (198%) | 14 | 62,568,341 | 381 | 3.045 (304%) |
| 2 | 248,966,461 | 976 | 1.960 (196%) | 15 | 80,783,214 | 365 | 2.259 (226%) |
| 3 | 223,996,068 | 972 | 2.170 (217%) | 16 | 71,693,149 | 278 | 1.939 (194%) |
| 4 | 119,216,639 | 415 | 1.741 (174%) | 17 | 72,251,135 | 331 | 2.291 (229%) |
| 5 | 107,836,144 | 452 | 2.096 (210%) | 18 | 68,494,538 | 335 | 2.445 (245%) |
| 6 | 116,888,256 | 342 | 1.463 (146%) | 19 | 60,445,663 | 313 | 2.589 (259%) |
| 7 | 100,009,711 | 384 | 1.920 (192%) | 20 | 51,049,468 | 312 | 3.056 (306%) |
| 8 | 90,615,088 | 311 | 1.716 (172%) | 21 | 49,987,992 | 261 | 2.611 (261%) |
| 9 | 94,583,238 | 358 | 1.893 (189%) | 22 | 50,780,147 | 215 | 2.117 (212%) |
| 10 | 86,377,204 | 342 | 1.980 (198%) | 23 | 62,282,865 | 514 | 4.126 (413%) |
| 11 | 62,170,480 | 383 | 3.080 (308%) | 24 | 41,976,827 | 11 | 0.131 (13.1%) |
| 12 | 79,028,859 | 408 | 2.581 (258%) | 25 | 45,223,504 | 220 | 2.432 (243%) |
| 13 | 82,951,069 | 511 | 3.080 (308%) | 26 | 44,047,080 | 85 | 0.965 (96.5%) |

*Chrom, chromosome number; Size, chromosome size; SNPS, number of SNPs on a given chromosome; *Coverage, percent of chromosome covered based on unevenly distributed SNPs.*

IV. Biological processes, functions, and disease term associations for candidate genes. Process and function data were retrieved from Princeton University’s Gene Ontology web platform (https://go.princeton.edu/cgi-bin/GOTermMapper; Boyle et al., 2004; Gene Ontology Consortium 2004) using the human (*Homo sapiens*) and mice (*Mus musculus*) genomes as references. Human disease terms associated with each gene were derived from Gene2Function using human as the reference species (http://www.gene2function.org; Hu, Comjean, Mohr, & Perrimon, 2017).

| **Gene** | **Orthologs** | **Process** | **Function** | **Disease term (humans)** |
| --- | --- | --- | --- | --- |
| *GHSR* | *Homo sapiens*  *Mus musculus* | anatomical structure development; reproduction; signal transduction; cell death; cellular component assembly; lipid metabolic process; response to stress; transport; cell-cell signaling; locomotion; biosynthetic process; nervous system process; cytoskeleton organization; anatomical structure formation involved in morphogenesis; small molecule metabolic process; cell proliferation; growth | - | growth hormone deficiency; height |
| SPATA16 | *Homo sapiens*  *Mus musculus* | anatomical structure development; reproduction; cell differentiation | - | spermatogenic failure |
| ECT2 | *Homo sapiens*  *Mus musculus* | anatomical structure development; cell differentiation; signal transduction; cell death; cellular component assembly; response to stress; transport; protein-containing complex assembly; mitotic cell cycle; chromosome segregation; cell morphogenesis; cell junction organization; cellular protein modification process; nucleocytoplasmic transport; cell division; cell cycle | enzyme regulator activity; enzyme binding | obesity-related traits |
| NCEH1 | *Homo sapiens*  *Mus musculus* | lipid metabolic process; catabolic process | - | obesity-related traits |
| TNFSF10 | *Homo sapiens*  *Mus musculus* | anatomical structure development; reproduction; signal transduction; cell death; cell-cell signaling; mitochondrion organization; immune system process | ion binding | - |
| FNDC3B | *Homo sapiens*  *Mus musculus* | - | RNA binding | height; intraocular pressure, corneal structure, Parkinson’s disease, heart rate, triptolide cytotoxicity |
| SEMA6D | *Homo sapiens*  *Mus musculus* | anatomical structure development; cell differentiation; locomotion; cell motility | - | coronary artery calcification; post bronchodilator FEV1/FVC ratio; diisocyanate-induced asthma; body mass index; eating disorder; carotid plaque burden; hair greying; response to radiotherapy in cancer (late toxicity) |

V. Parameter value estimates from the Bayesian sparse linear mixed model (BSLMM). Convergence was attained for most parameters (PVE, PGE, rho, and h). Lack of convergence and large uncertainty in n-gamma and pi parameters are likely the result of small sample sizes. Parameters were sampled every 10 iterations, thus each plot has 500,000 data points (5 million iterations total).


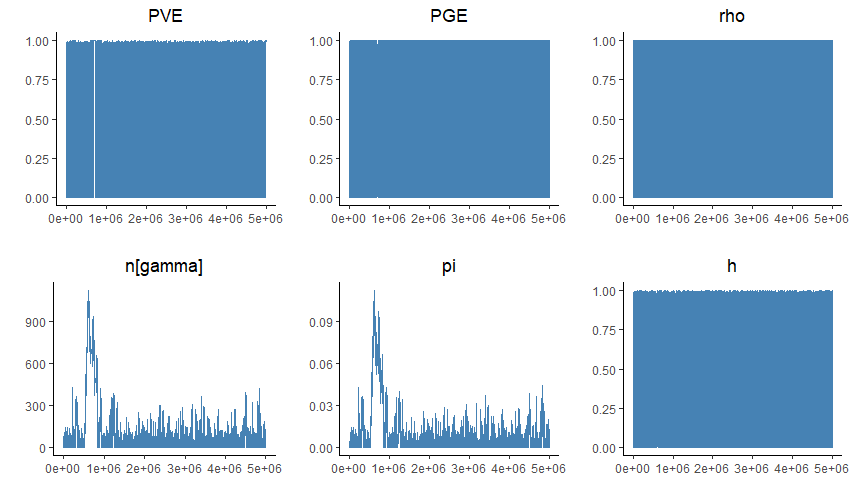


References

Boyle, E. I., Weng, S., Gollub, J., Jin, H., Botstein, D., Cherry, J. M., & Sherlock, G. (2004). GO::TermFinder—open source software for accessing Gene Ontology information and finding significantly enriched Gene Ontology terms associated with a list of genes. *Bioinformatics, 20*(18), 3710-3715. doi:10.1093/bioinformatics/bth456

Hu, Y., Comjean, A., Mohr, S. E., & Perrimon, N. (2017). Gene2Function: An Integrated Online Resource for Gene Function Discovery. *G3: Genes|Genomes|Genetics, 7*(8), 2855. doi:10.1534/g3.117.043885
